# Temporal Fluctuation of Mood in Gaming Task Modulates Feedback Negativity: EEG Study With Virtual Reality

**DOI:** 10.3389/fnhum.2021.536288

**Published:** 2021-06-03

**Authors:** Yusuke Yokota, Yasushi Naruse

**Affiliations:** Center for Information and Neural Networks (CiNet), National Institute of Information and Communications Technology and Osaka University, Kobe, Japan

**Keywords:** virtual reality, electroencephalogram, feedback negativity, reward positivity, real world recording, linear mixed effect model, mood

## Abstract

Feedback outcomes are generally classified into positive and negative feedback. People often predict a feedback outcome with information that is based on both objective facts and uncertain subjective information, such as a mood. For example, if an action leads to good results consecutively, people performing the action overestimate the behavioral result of the next action. In electroencephalogram measurements, negative feedback evokes negative potential, called feedback negativity, and positive feedback evokes positive potential, called reward positivity. The present study investigated the relationship between the degree of the mood caused by the feedback outcome and the error-related brain potentials. We measured the electroencephalogram activity while the participants played a virtual reality shooting game. The experimental task was to shoot down a cannonball flying toward the player using a handgun. The task difficulty was determined from the size and curve of the flying cannonball. These gaming parameters affected the outcome probability of shooting the target in the game. We also implemented configurations in the game, such as the player’s life points and play times. These configurations affected the outcome magnitude of shooting the target in the game. Moreover, we used the temporal accuracy of shooting in the game as the parameter of the mood. We investigated the relationship between these experimental features and the event-related potentials using the single-trial-based linear mixed-effects model analysis. The feedback negativity was observed at an error trial, and its amplitude was modulated with the outcome probability and the mood. Conversely, reward positivity was observed at hit trials, but its amplitude was modulated with the outcome probability and outcome magnitude. This result suggests that feedback negativity is enhanced according to not only the feedback probability but also the mood that was changed depending on the temporal gaming outcome.

## Introduction

Humans require feedback to evaluate their own actions, regardless of whether they have resulted in good outcomes or bad. If the outcome is good, we can continue the action, but if the outcome is bad, we must modify the action. Many cognitive neuroscience studies have focused on feedback. Feedback evokes neural activities, which are measured as event-related potentials (ERPs) using an electroencephalogram (EEG).

A common ERP related to feedback is feedback negativity (FN). FN is a front-central ERP component and is strongly observed between 200 and 350 ms after feedback is received (Miltner et al., 1997; Gehring and Willoughby, 2002; Hajcak et al., 2007). FN has often been observed in money-rewarding tasks. Many studies have revealed how FN is modulated with experimental features such as outcome valence (positive vs. negative), magnitude (large vs. small), and probability (high vs. low). Traditionally, a stronger FN is evoked with negative feedback compared to positive (Miltner et al., 1997; Gehring and Willoughby, 2002; Yeung and Sanfey, 2004; Hajcak et al., 2007). Several studies found that unexpected negative feedback evokes greater FN than expected feedback (Holroyd and Coles, 2002; Nieuwenhuis et al., 2002; Potts et al., 2006; Walsh and Anderson, 2011). The relationship between the outcome magnitude and FN modulation is currently under debate. Several studies have argued that FN is not sensitive to magnitude (Yeung and Sanfey, 2004; Hajcak et al., 2006, 2007), while other studies have reported that FN modulates both the outcome valence and magnitude (Goyer et al., 2008; Wu and Zhou, 2009; Bellebaum et al., 2010; Kreussel et al., 2012).

Many studies have discussed FN modulation from the differences in waveforms between positive and negative feedbacks. From here on, we defined the negative potential generated by the differential waveform between negative deflection after a negative feedback and positive deflection after a positive feedback as differential feedback negativity (dFN). Recently, several studies have argued that the amplitude of dFN is not directly attributed to the negative potential caused by negative feedback (Cohen et al., 2007; Holroyd et al., 2008; Foti et al., 2011a; Walsh and Anderson, 2012). One report hypothesizes that unexpected feedback evokes negative potential as N200 regardless of whether the outcome valence is positive or negative, and expected positive feedback evokes a positive potential called reward positivity (RewP) (Holroyd et al., 2008; Foti et al., 2011a; Walsh and Anderson, 2012). As a result, the positive deflection attenuates the N200. Traditionally, a stronger RewP is caused by improbable positive (reward) feedback rather than probable positive feedback, and these modulations were more sensitive than instances with negative feedback (Cohen et al., 2007; Walsh and Anderson, 2012; Sambrook and Goslin, 2014, 2016). Furthermore, RewP has been reported to be related with reward magnitude (Cherniawsky and Holroyd, 2013; Qu et al., 2013). Therefore, the negative potential evoked by negative feedback (e.g., loss-related FN) and RewP is considered to be caused by different neural mechanisms. Although it is common to define FN as the difference between positive and negative waveforms, several recent literatures have reported various methods to verify the FN components (Gheza et al., 2018; Paul et al., 2020). Although the mechanisms of these ERPs are currently under debate, we should discuss the modulation not only of the dFN but also of the negative potential evoked by negative feedback and the RewP evoked by positive feedback as independent ERP components. Although “FN” has been treated in previous studies as ERPs that were the difference in waveforms between loss and gain feedbacks or were the negative potential evoked by negative feedback, we treated the negative potential evoked by the negative feedback as FN in this article.

We investigated FN/RewP from two perspectives. Firstly, our study focused on mood related to feedback outcome. Several previous studies reported a relationship between the mood’s influence and FN/RewP modulation. The definition of the keyword “moods” differed in each previous study. For example, several previous studies have used emotional pictures or an imagery procedure method to induce a negative or positive mood in their participants (Foti and Hajcak, 2010; Foti et al., 2011b; Bakic et al., 2014; Angus et al., 2015; Riepl et al., 2016; Paul and Pourtois, 2017; Bandyopadhyay et al., 2019). In other words, the moods were controlled to a specific valence (positive, negative, or neutral) by the experimenter. Some studies have also modulated participants’ positive moods by controlling the magnitudes of feedback reward (Meadows et al., 2016; Paul et al., 2020). A previous study defined a mood that is linked to specific events and reflects the cumulative impact of multiple stimuli, while an emotion typically relates to a single stimulus (Eldar et al., 2016). They also reported the relationship between a series of feedback outcomes and the mood changes. The errors in positive moods result in large prediction errors. In contrast, the accumulation of negative outcomes decreases the mood of participants. This mood might change according to the success and the failure rates in past trials. The temporal mood during the task constantly fluctuates depending on each feedback outcome in the task. Based on this definition, the manipulation of moods through emotional pictures or memory recall employed in the previous study was more of a manipulation of the participants’ “emotion,” not the “mood.” Thereafter, the moods in the present study are followed in accordance with the definition by Eldar et al. (2016). Secondly, we focused on the characteristics of the experimental task that generate positive and negative feedbacks. A lot of the previous studies discussed, in terms of positive/negative feedbacks derived from the gambling task, choosing one of multiple options. In most of the previous experiments, the feedback probabilities were determined by the experimental conditions. In other words, participants expected the upcoming outcomes according to the probabilities determined by a computer program. In this case, the participants did not fundamentally have the responsibility for the outcome of their choice since the outcome depended entirely on the probability. We used a task in which actions according to one’s skills lead to good or bad outcomes and focused on the mood that occurred in the tasks that involved responsibility for the outcome of one’s actions. The novelty of this study is that we analyzed such temporal fluctuations of moods that occurred during tasks that involve responsibility for the outcome of one’s actions. No research has reported an investigation of the relationship between the continuous temporal fluctuations of mood and the ERP modulations.

In this study, we experimented with a gaming task to make participants more aware of the temporal fluctuations of mood that occur in tasks that involve responsibility for the outcome of one’s actions. Because the gaming tasks increase the participants’ intrinsic motivation, we expected that the mood would enhance the feedback-related ERPs. We developed an original virtual reality (VR) shooting game as an experimental task. Although VR is generally used for entertainment, it is an excellent technology for experimental tasks. For example, VR technology was widely used in the field of neurorehabilitation because it allows participants to feel deeply immersed in a virtual environment and motivates them for the tasks (Adamovich et al., 2009; Teo et al., 2016). In particular, the integration of VR technology and tasks of adaptive computer games provides a rich and challenging training environment and a useful rehabilitation tool for children (Brutsch et al., 2010; Demers et al., 2020). VR contents are also effective tools for EEG recordings. Previous studies have confirmed that VR contents can be used as emotional induction tools (Riva et al., 2007; Banos et al., 2012; Herrero et al., 2014) and enhanced the intensity of emotions as well as the sense of presence compared to non-VR contents (Chirico et al., 2017). Other studies showed that performance enhancement experiments using EEG in a VR environment may lead to better results compared to non-VR experiments (Slobounov et al., 2015; Ko et al., 2020). VR technology is able to serve as an experimental environment that is difficult to simulate in the real world. Furthermore, because the VR environment is controlled by a computer program, we can acquire all event onsets in the task with a millisecond resolution. The present study investigated the correlation of ERP amplitude (FN and RewP) and experimental features (VR events and mood) at a single-trial level.

## Materials and Methods

### Participants

Thirty-four participants (18 males, 16 females, age range = 21–54 years) participated in this study. All participants had normal hearing and normal or corrected-to-normal vision. Participants provided informed written consent after the details of the procedure had been explained and before the experiment. All experimental procedures were approved by the Ethical Committee for Human and Animal Research of the National Institute of Information and Communications Technology. All experiments were performed in accordance with the ethical standards described in the Declaration of Helsinki.

### Experimental Procedure

**FIGURE 1:**
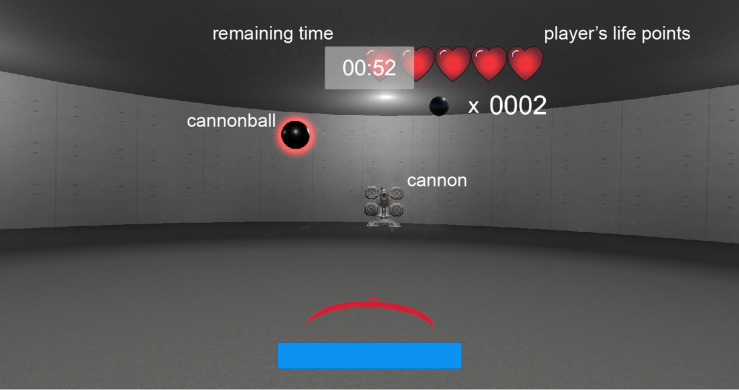
Screenshot of the virtual reality (VR) shooting game. Participants were instructed to shoot down cannonballs using a handgun in a VR environment.

We used an original VR shooting game as an experimental task. A screenshot of the shooting game is shown in Figure 1, and a movie of the game is shown in Supplementary Video 1. In the game, a cannon was placed in front of a participant and fired cannonballs toward the participant. The participants were instructed to shoot down the cannonballs using a handgun. Participants were allowed to fire only once at each cannonball. The cannonballs flew toward the player with a straight or a parabolic path from the cannon. If the participants failed to shoot down the cannonball, they were bombarded with the cannonballs and lost one life point in the game stage. When the cannonball was located within approximately 30% distance from cannon to the player, its color was changed from black to gray. In this case, the cannonball did not disappear, even if it was hit by a player’s shot. We defined this feedback result as an infrangible hit event. This was designed to inhibit the player from firing bullets at a close range. However, we excluded this analysis of the feedback outcomes from the EEG analysis because it was not the main objective of the present study. The playing time for each level was 1 min, and a participant started with five life points in each level. The participants were instructed to survive for 1 min without losing all of their life points and were also instructed to complete as many stage levels as possible. The game stages consisted of 13 levels. Parameters for the maximum and minimum cannonball sizes and curve trajectories were set for each level. The size and the curve trajectory of the cannonballs for each trial were randomly selected between the maximum and minimum range in each level. The game became harder as it progressed through the levels. Specifically, the cannonball’s size became smaller and the curve of the trajectory became tighter. All the participants practiced for 5 min before the EEG experiment. They were instructed to restart the game from the beginning of the stage if they lost all life points in each level. Consequently, the player could play the shooting game at a suitable difficulty. The participants sat in a chair and held the VR controller in their dominant hand to play the game.

### Experimental Devices

**FIGURE 2:**
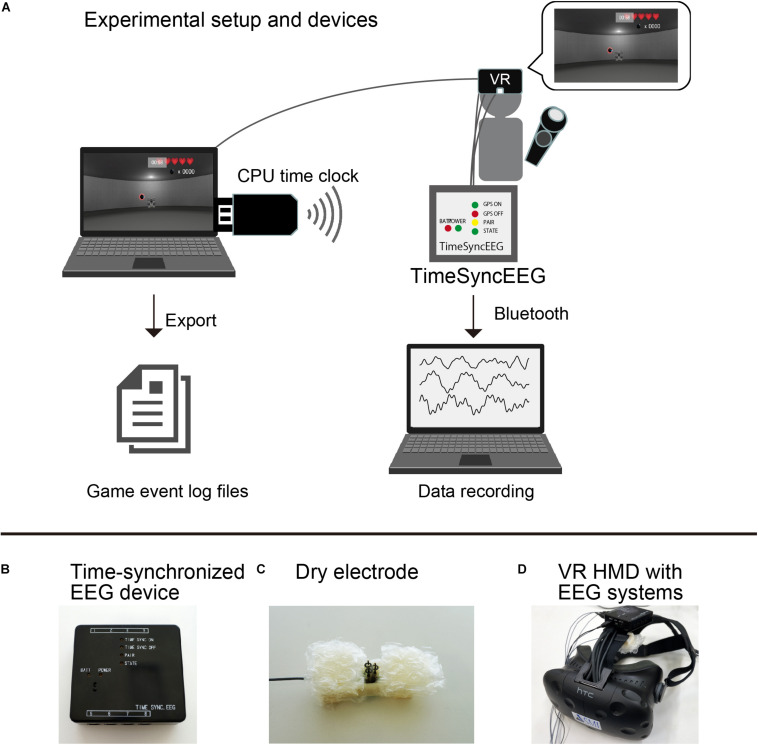
**(A)** Experimental setup and devices. **(B)** Time-synchronized electroencephalogram (EEG) device. **(C)** Dry electrode. **(D)** Virtual reality (VR) head-mounted display with an EEG recording system.

An illustration of the experimental setup is shown in Figure 2A. Our developed VR software was able to export the gaming log to a file. The gaming log included the participant’s position, the cannonball’s size, cannonball’s curve trajectory, the cannonball’s position, and any game event onset time such as error (a failed shot) and hit (a successful shot) events. These parameters were exported for every video frame. Furthermore, the time of each frame based on the CPU time of a computer that ran the software was added to the log file. In parallel, the CPU time was transmitted to the EEG device using Bluetooth with a time precision of microseconds. All sampling data recorded by the EEG device were linked to the CPU time of that computer. We analyzed ERPs related to the game events in the VR software based on the CPU time.

Illustrations of the experimental devices are shown in Figures 2B–D. The present study used HTC Vive (HTC, New Taipei, TWN) for the VR experience. The participants wore a head-mounted display to observe images in the VR space and played the game with a controller device using their dominant hand. They could shoot the bullet by pressing the trigger button on the controller device. The frame rate of the VR head-mounted display was 90 Hz. To record the participants’ EEG activities while they wore the head-mounted display, we set an electrode socket on the top band of the head-mounted display. We attached a dry electrode (Unique Medical, Japan) to the electrode socket and measured the EEG activities at the FCz position according to the international 10–20 system. EEG data were measured by one electrode using an original eight-channel portable EEG device capable of supporting the CPU time [Miyuki Giken, original development based on Polymate Mini AP108 (*W* × *D* × *H*: 52 × 50 × 20 mm, 80 g), Japan]. To measure the electrooculograms (EOGs), two electrodes were placed on the top and the side of the right eye of each participant. All recorded signals were referenced to the left mastoid, and the ground electrode was placed on the right mastoid. The EEG and EOG data were sampled at 500 Hz.

### EEG Data Analyses

**FIGURE 3:**
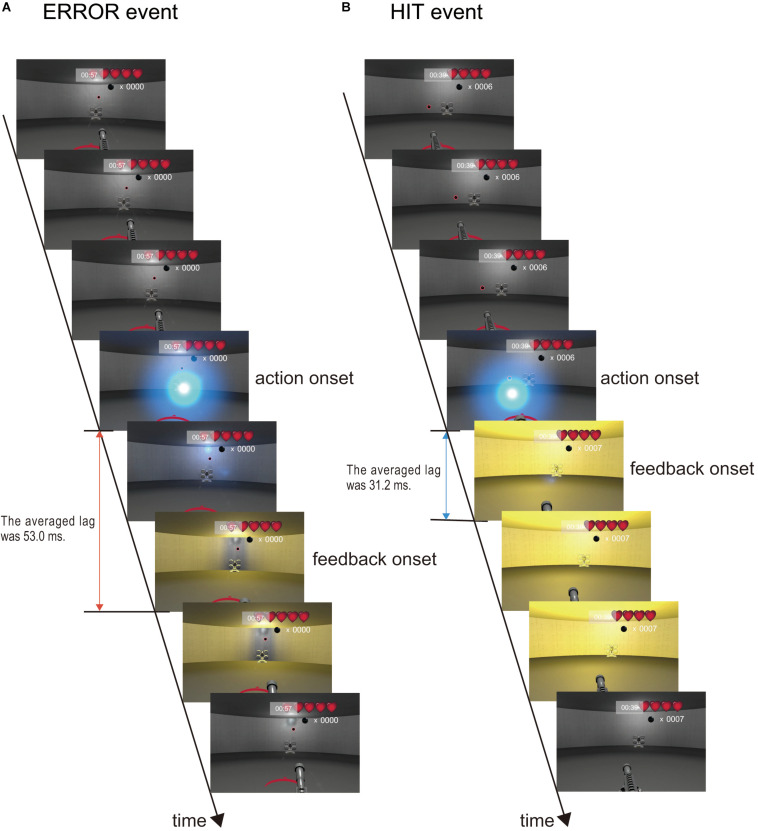
Criteria for two event onsets. We focused on the two game events. The *blue sphere* was the bullet fired by the participants. **(A)** ERROR event. The bullet fired by the participants flew in a direction different from the position of the cannonball. The ball hit the wall and the cannonball did not disappear. The scene appears in approximately 3 s, as shown in Supplementary Video 1. **(B)** HIT event. The bullet fired by the participants flew into position on a cannonball. The ball hit the cannonball, and the cannonball disappeared. The scene appears in approximately 21 s in Supplementary Video 1.

Electroencephalogram analyses were performed using MATLAB 2017b (MathWorks, Inc., Natick, MA, United States). A digital finite impulse response bandpass filter (1–15 Hz, order 2,500) was applied to the continuous EEG data. Subsequently, we used the automated artifact removal method to remove eye movement related artifacts from the EEG data (Schlogl et al., 2007). The EEG data were divided into 2,000-ms epochs (-1,000 to +1,000 ms) based on the two event onsets. An illustration of the two event onsets is shown in Figure 3. The blue sphere was the bullet fired by the participants and indicated the action onset. The yellow flash indicated the feedback onset. One game event was ERROR. The ERROR is an event in which the player shot a bullet, but did not hit a cannonball (Figure 3A and Supplementary Video 1). The ERROR event onset is the moment the bullet hit a wall in the playing field. The delay between the action onset and the feedback onset for ERROR was 53.0 ms (*SD* = 1.59). Another event was HIT, which is when the player successfully shot down a cannonball (Figure 3B and Supplementary Video 1). The delay between the action onset and the feedback onset for HIT was 31.2 ms (*SD* = 1.86). These two event onsets were the feedback moments of each event and not the participant’s shoot moment. Figure 3 was made by a series of frame images extracted from Supplementary Video 1. Note that the frame rate of Supplementary Video 1 was 30 frames per second, but the actual frame rate of the VR head-mounted display was 90 frames per second. Baseline correction was performed using the averaged amplitude from −1,000 to −500 ms.

### Automatic Artifact Rejection With Threshold

The purpose of the threshold-based artifact rejection is to improve the signal-to-noise ratio of the averaged EEG dataset by removing the trials that include large noise. The unsynchronized noise between trials is attenuated according to the root of the number of the trials using the averaging method. Therefore, we must employ a suitable threshold for each participant to maximize the signal-to-noise ratio of the averaged EEG data.

We proposed a formula to minimize the noise in the averaged data. Here, we denoted the entire EEG data matrix as ***D*** with *N* trials and *T* time series. *N* includes all HIT and ERROR event trials. Then, the data matrix composed of good EEG trials whose maximum value in the time series did not exceed a threshold *τ* was denoted as ***D***_τ_. The ***D***_τ_ matrix consisted of the number of data trials. Next, we recreated the data vector Reshaped.***D***_τ_ by connecting the trials along the time series dimension of the data matrix ***D***_τ_. The vector length of the Reshaped.***D***_τ_ was *N*_***D***_τ__^∗^ *T*, where *N*_***D***_τ__ is the number of trials in ***D***_τ_. Here, we defined the noise of the data as the standard deviation of Reshaped.***D***_τ_. Although the recorded EEG data contain ERP activities, we made the assumption that the more fluctuations in the recording signal, the more noise are included since the ERP activity is constant across all the trials. Thus, we assumed that the std(Reshaped. ***D***_τ_) is the amount of noise in the single trials in ***D***_τ_, and $\frac{\text{std}\left(\text{Reshaped}.D_{\tau }\right)}{\sqrt{N_{D_{\tau }}}}$ is the amount of noise in the averaged EEG data from ***D***_τ_. Here, we determined the optimal threshold τ_opt_ for each participant as follows:

$$\tau_{\text{opt}}=\underset{\tau }{\text{argmin}}\frac{\text{std}\left(\text{Reshaped}.{\text{D}}_{\tau }\right)}{\sqrt{N_{D_{\tau }}}}$$

The thresholds τ were changed from 10 to 100 μV in 1-μV steps in order to determine τ_opt_, which minimizes the amount of noise in the averaged EEG data.

### Statistical Analyses

The present study employed an LME (linear mixed effects) for statistical analysis. In traditional neuroscience studies for event-related potentials, the EEG data are averaged for each experimental condition and participant; then, analysis of variance (ANOVA) is often used to test the statistical significance between the experimental conditions. This traditional technique is based on the hypothesis that all participants and the experimental condition average the same number of trials. However, because the number of trials in our study depended on each participant’s action results, they differed greatly for each participant. Therefore, it was not desirable to use the traditional ANOVA approach for the current VR experimental data. Because LME is robust for unequal number of observations for each experimental condition and participant (Fromer et al., 2018), we used an LME model instead of the traditional ANOVA.

The LME model allows estimation at a single trial level for objective variables. Each game event in the VR shooting has various gaming parameters that players could experience during the game, such as the size and curve of the cannonball, the playing time for each level, and the player’s remaining life points. The LME model could reveal the relationship between the game event defined by these game parameters and the corresponding ERPs.

**FIGURE 4:**
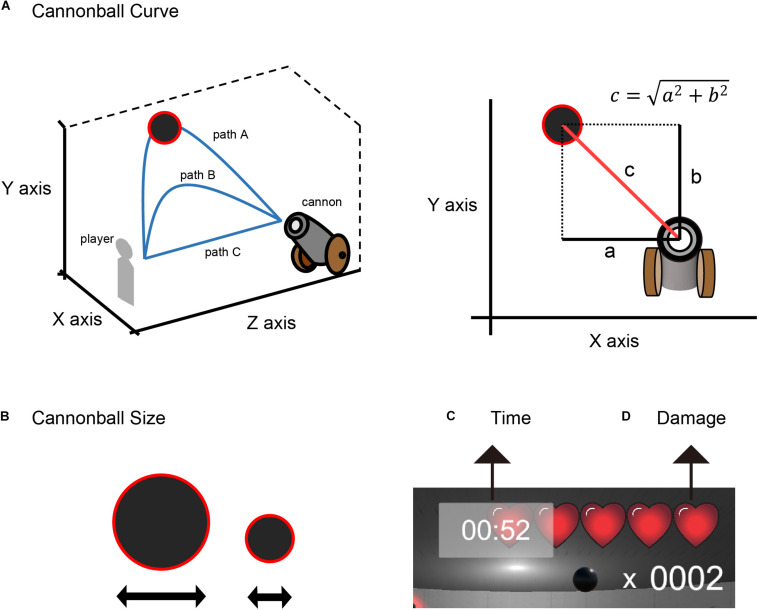
Experimental features. **(A)** cannonball curve, **(B)** cannonball size, **(C)** time, and **(D)** damage.

The LME model estimates the regression weights of the fixed and the random effects. We defined a corresponding value for the size and curve of the cannonballs (size and curve, respectively), a playing time (time), a damage count (damage) of each level, hit count of up to 10 events before (mood index), and the feedback outcome of a participant’s action (valence) as fixed effects. We defined a participant difference as a random intercept (participants). The larger the cannonball, the easier it was to shoot it down. The wider the curve, the easier it was to shoot the cannonball down. In this experiment, the curve trajectory of the cannonballs was determined by two parameters related to the horizontal and vertical axes. Here, we defined the three-dimensional coordinate space in VR, as shown in Figure 4A. The curve trajectory of the cannonballs was defined by the diagonal line $\text{c}=\sqrt{a^{2}+b^{2}}$, where *a* and *b* are the maximum separation distances of the cannonball in the *X*− and *Y*-axis, respectively, from the participant’s view.

The cannonball’s size and curve were parameters related to the difficulty of the shot. Therefore, these two parameters correspond to the size of the effect of outcome probability. The playing time and damage count do not affect the difficulty of the shot. Because the error of shooting with a few participant life points and shorter time remaining is a serious error, these parameters correspond to the size of the effect of outcome magnitude.

In general, participants would underestimate the accuracy of their next trial if they had excessive errors by then. On the other hand, participants would overestimate their accuracy for the next trial if they had successful hits by then. Therefore, in the present study, we defined the mood index as how well a player had completed shooting until the newest trial. It can be regarded as a temporal accuracy in the trial. We defined the mood index M(*n*) in trial *n* as:

$$\text{M}\,\left(n\right)=\sum_{t=1}^{T}h\,\left[n-t\right]\cdot \alpha^{t},$$

where *h*[*n* - *t*] represents the result of shooting in the *n* - *t* trial (hit: 1, error and infrangible hit: 0), *T* represents the decay constant, and α represents the decay coefficient. We used α = 0.8 and *T* = 10. Note that the first 10 trials in each game stage were discarded. The mood index corresponds to the size of the effect of the temporal fluctuation of the mood and changes continuously in each trial. If a player completely shot down all the cannonballs in the recent 10 trials, the mood index was at a maximum. If a player could not shoot any cannonballs in the recent 10 trials, the mood index was at a minimum. The mood index was not related to the difficulty of each trial. We used valence as a fixed effect to express a feedback outcome of the participant’s action, and it was a categorical variable with “0” for the ERROR event and “1” for the HIT event.

The single-trial ERP amplitudes for the LME analysis were obtained using the following procedures. Firstly, we calculated the average ERPs of the hit and error events for each participant. Secondly, the negative peak latencies, between 150 and 250 ms after feedback in the averaged waveform of the error event, were obtained for each participant. Similarly, the positive peak latencies, between 150 and 250 ms after feedback in the averaged waveform of the hit event, were obtained for each participant. Finally, the averaged amplitude of 25 ms before and after the peak latencies in each participant and event was considered the single-trial ERP amplitude.

Our initial LME model described in Wilkinson notation is as follows: single-trial ERP amplitude ∼ (size ^∗^ curve + time ^∗^ damage + mood index) ^∗^ valence + (1| participants). All fixed effects were transformed into *z*-scores. To avoid multicollinearity, we calculated the variance inflation factor (VIF) of each fixed effect and the interactions. The predictors with a VIF value greater than 10 were excluded from the initial model. We used R version 3.5.1 for statistical analysis and performed an LME analysis using the *lme4* and the *lmerTest* package. We used the *anova* function of the *lmerTest* package to perform type III analysis of variance for the LME model. We used Satterthwaite’s method to compute the denominator degrees of freedom and *F*-statistics.

We investigated the effects of task difficulty parameters on behavioral outcomes (HIT or ERROR) in a shooting game. The model described in Wilkinson Notation is as follows: behavioral outcomes ∼ size ^∗^ curve + (1 | participants). We performed a general linear mixed model analysis using the *lme4* package. The objective variable was set as a categorical variable with an HIT of 1 and an ERROR of 0. Therefore, the probability distribution was a binomial distribution.

## Results

### Behavioral Analysis

The averaged hit, error, and infrangible hit rates across participants were 61.6% (*SD* = 0.0659), 33.4% (*SD* = 0.0847), and 5.00% (*SD* = 0.0395), respectively. The behavioral outcome model estimates are summarized in Table 1. Size showed a positive correlation with the behavioral outcome. Thus, the results showed that the HIT outcomes were dependent on the size of the cannonball.

**TABLE 1:** Effects of behavioral outcomes.

**Variable**	**Estimate**	**SE**	***z*-value**	***p* value**
Intercept	0.755	0.0765	9.86	<0.001
Size	0.117	0.0233	5.01	<0.001
Curve	−0.00349	0.0190	−0.183	0.854
Size:curve	0.0393	0.0207	1.90	0.0575

**Variance components**	**SD**	**Goodness of fit**

Participants	0.429	Log likelihood	−8181.2	
		REML deviance	16362.5	

The averaged reaction times of hit, error, and infrangible hit were 1.04 s (*SD* = 0.175), 1.02 s (*SD* = 0.179), and 1.47 s (*SD* = 0.178), respectively. The averaged reaction time after a hit trial was 1.01 s (*SD* = 0.184), and the averaged reaction time after an error or an infrangible hit trial was 1.14 s (*SD* = 0.179). The *t* test showed a significant difference between these reaction times (RTs) [*t*(33) = 11.3, *p* < 0.001, *r* = 0.89]. Therefore, the results showed that the RT after an error or an infrangible hit trial was significantly slower than the RT after a hit trial. The average number of game restarts for the participants was 38 times (*SD* = 9.99).

### EEG Analysis

**FIGURE 5:**
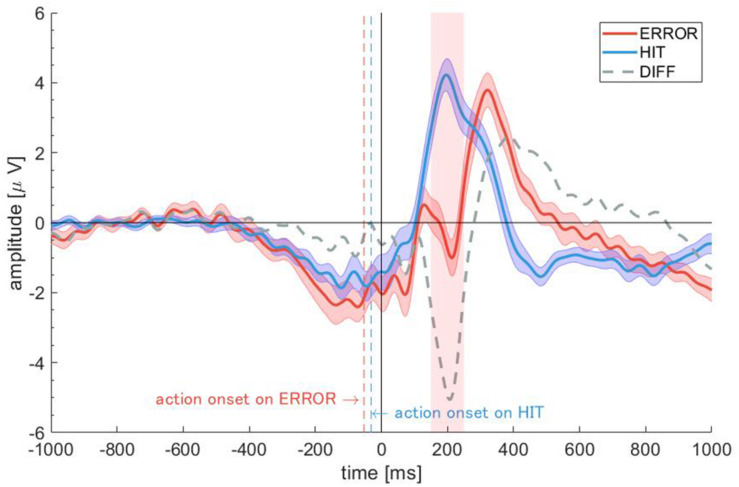
Grand averaged event-related potentials (ERPs) for the ERROR and HIT events. The *shades around the waveforms* indicate the standard errors among the participants.

The optimal threshold for artifact rejection of each participant and the remaining trials are shown in Supplementary Figures 1, 2. The averaged optimal threshold across participants was 39.9 (*SD* = 15.5). As a result of artifact rejection, the average numbers of trials across all participants in the hit and error events were 315 (*SD* = 79.5) and 170 (*SD* = 59.1) trials, respectively. The grand averaged ERPs for each event (HIT and ERROR) and the difference between the events are shown in Figure 5. The blue dashed line indicates the action onset of the HIT. It also indicates the action onset of the ERROR. The shades around the waveforms indicate the standard error among the participants. For the ERROR event, we confirmed a negative potential after event onset (from +200 to +250 ms). Thus, we defined the negative potential in the ERROR event as the FN. For the HIT event, we confirmed a large positive potential after event onset (from +200 to +250 ms). We defined the positive potential in the HIT event as the RewP. The shades of ERP waveforms represent the standard errors over the participants.

Because the VIF for the interaction of time and valence was greater than 10, we excluded this interaction from the initial model that we have described above. The conducted model was as follows: ERPs ∼ (size ^∗^ curve + damage + mood index) ^∗^ valence + time + time:damage + time:damage:valence + (1| participants). The VIF of all the fixed effects and the interactions was less than 10. All variables were transformed into *z*-scores.

The type III analysis of variance table for the model is summarized in Table 2. Analysis of variance revealed main effects (size, curve, and valence) and significant interactions (curve:valence, mood index:valence, and time:damage:valence) (see Table 2 for statistics). The predictor of valence showed a significant difference between the ERROR and HIT events. The predictors of size and curve showed significant differences, indicating that both predictors affected the amplitude of FN and RewP commonly. The three interactions including valence showed significant coefficients. The curve, the mood index, and the interaction of time and damage belonged to the outcome probability, mood, and outcome magnitude, respectively. The results indicate that these predictors have significantly different effects on FN and RewP. The estimates and statistics of each predictor for the ERROR and HIT events are shown in Tables 3, 4, respectively. We found significant coefficients of size, curve, and mood index in the ERROR event. The curve showed a positive correlation with FN and the size showed negative correlations with FN. Thus, the results showed that the error against a smaller curve and a larger size of the cannonballs evoked a larger negative FN amplitude. In other words, the error of shooting in an easy situation evoked a larger negative FN amplitude. The mood index showed a negative correlation, suggesting that the error in good mood evoked a larger negative FN amplitude. We found significant coefficients of size, curve, and interaction of time and damage in the HIT event. RewP was positively correlated with curve and was negatively correlated with size. The interaction between playing time and damage count showed a positive correlation with RewP. These results indicate that shooting at a high difficulty and under severe pressure resulted in a larger RewP.

**TABLE 2:** Type III analysis of variance with Satterthwaite’s method for the linear mixed-effects (LME) model.

**Variable**	**Sum of squares**	***F* value**	***p* value**	**Partial *η*^2^**
Size	15.8	18.9	<0.001	0.00190
Curve	100.3	120.2	<0.001	0.00910
Time	0.01	0.0156	0.900	0.00000119
Damage	1.22	1.46	0.227	0.000112
Mood index	0.97	1.17	0.280	0.0000894
Valence	942.4	1129.2	<0.001	0.08
Size:curve	0.65	0.775	0.379	0.0000593
Time:damage	1.22	1.46	0.227	0.000112
Size:valence	0.09	0.112	0.738	0.00000856
Curve:valence	10.6	12.7	<0.001	0.000969
Damage:valence	0.03	0.0411	0.839	0.00000314
Mood index:valence	14.1	16.9	<0.001	0.00129
Size:curve:valence	0.29	0.342	0.559	0.0000262
Time:damage:valence	4.20	5.04	0.0248	0.000385

**TABLE 3:** Effects of feedback negativity (FN) amplitude.

**Variable**	**Estimate**	**SE**	***t* value**	***p* value**
Intercept	–0.526	0.0339	–15.5	<0.001
Size	–0.0541	0.0188	–2.88	<0.01
Curve	0.0763	0.0180	4.25	<0.001
Time	–0.0124	0.0154	–0.805	0.421
Damage	0.00169	0.0135	0.125	0.901
Mood index	–0.0497	0.0160	–3.11	<0.01
Size:curve	–0.0163	0.0198	–0.825	0.409
Time:damage	–0.0195	0.0161	–1.21	0.227

**TABLE 4:** Effects of reward positivity (RewP) amplitude.

**Variable**	**Estimate**	**SE**	***t* value**	***p* value**
Intercept	0.256	0.0297	8.63	<0.001
Size	–0.0472	0.0113	–4.18	<0.001
Curve	0.149	0.00992	15.0	<0.001
Time	0.00169	0.0135	0.125	0.900
Damage	–0.0159	0.0135	–1.18	0.239
Mood index	0.0239	0.0137	1.74	0.0815
Size:curve	–0.00331	0.0103	–0.323	0.747
Time:damage	0.0247	0.0110	2.24	0.0250

## Discussion

The present study used an original VR shooting game as an experimental task and measured the brain EEG activity while participants played the game. We focused on two kinds of ERPs related to negative and positive feedbacks. The large FN was observed at the frontocentral electrode during the 200 ms after negative feedback. RewP was observed at the frontocentral electrode during the 200 ms after positive feedback.

For FN, all fixed effects that were significant belonged to outcome probabilities and mood, and the fixed effects corresponding to outcome magnitude were not significant. Therefore, these results suggest that FN is sensitive to outcome probability and is not affected by outcome magnitude. This finding was consistent with previous studies (Yeung and Sanfey, 2004; Hajcak et al., 2006, 2007). We suggested that the negative potential of FN is modulated not only by the outcome probability but also by the mood, which is the participant’s temporal mental state.

The coefficients of the curve in RewP were larger than those in FN. The significant interaction of the curves and valence in Table 2 would reflect the difference. The size and curve of the cannonball belonged to the outcome probability of feedback. The playing time and damage count belonged to the outcome magnitude of feedback. The single predictors of time and damage were not significant, but the absolute value of the coefficients of these interactions was larger than that of the coefficients of each predictor. This result indicates that, with the passage of time, the error event was recognized as a serious failure for the participants. Recent reports have shown that RewP is sensitive to the outcome magnitude (Paul et al., 2020), and the present results support these findings. On the other hand, unlike FN, the RewP did not show significant statistics related to the mood.

These suggest that processes underlying the generation of the FN and RewP might have different mechanisms since the FN was modulated not only by the outcome probability but also by the mood. Although the mood is constantly changing depending on the series of feedback results, it is interesting to note that mood affects only the FN modulation. Neural processing of the errors occurs because of the integration of complex information processing in the brain. Moreover, neural processing of the errors is modulated by individual differences as subjective factors. Several studies have reported that error-related brain responses are enhanced by various individual differences [e.g., decisiveness (Senderecka et al., 2018), conscientiousness (Pailing and Segalowitz, 2004), socialization (Dikman and Allen, 2000), and agreeableness (Tops et al., 2006)]. The present study showed the novel findings that error-related brain responses are changed not only by the individual differences due to subjective factors but also by the temporal fluctuations of the mood for the upcoming outcome of the task.

There are several limitations of this study. Firstly, it was difficult to distinguish whether the positive potential in the hit event was RewP or other kinds of ERPs (e.g., shifted P300 or P2). The latency of positive deflection for a hit was 200 ms. Moreover, the positive deflection appears to be formed by a bimodal peak. For these reasons, we considered the positive peak at the 200 ms to be RewP that superimposed on P300. Next, we considered the possibility whether the positive peak around 200 ms after visual feedback was P2 (P200). In general, a series of early visual ERP components such as N1, P1, and P2 are evoked in response to visual stimuli (Keil et al., 2002; Batty and Taylor, 2003; Carretié et al., 2004; Foti et al., 2009). Emotional images appear to impact the magnitude of P1 (Olofsson and Polich, 2007; Mueller et al., 2009), and N1 is sensitive to the emotional content of visual stimuli (Carretié et al., 2007; Foti et al., 2009). Following N1, P2 is evoked at approximately 180 ms after stimulus onset (Carretié et al., 2004). The P2 amplitude is enhanced for infrequent target stimuli than for frequent standard stimuli, suggesting that P2 indexes selective attention (Luck and Hillyard, 1994). However, in the present study, the HIT event was a frequent feedback outcome compared to the ERROR event. Therefore, we considered the positive peak in positive feedback at 200 ms to be RewP, not P2. The present ERPs in positive feedback differed from the classical ERP waveforms. We believe that this is due to the fact that the present study used a task that involved responsibility for the outcome of one’s actions. In fact, a previous study that used a task involving responsibility for the outcome of one’s actions observed similar ERP waveforms to the present study in positive feedback (Joch et al., 2017). The differences in the task characteristics between our task and the classical gambling task may have contributed to the generation of different waveforms. There is a possibility that the RewP and other ERPs (shifted P300 and P2) are distinguished from multichannel EEG data. Moreover, the negative and positive potentials evoked at 200–300 ms represent brain activities related to human consciousness (Rohaut and Naccache, 2017; Hermann et al., 2020). EEG recordings with more electrodes allow for rich spatiotemporal ERP analysis and can elucidate the brain mechanisms from various perspectives. In fact, a previous study performed a spatiotemporal mapping analysis called topographical analysis to FN and RewP using 64 channels of EEG data (Gheza et al., 2018). These analyses will give us sophisticated data related to mood and emotion. However, it was difficult to place a large number of channels on the scalp while the participants wore a VR head-mounted display. Therefore, we placed an electrode at FCz, where FN/RewP is strongly measured. The small number of measurement electrodes in EEG in this experiment is the second limitation. Thirdly, there was a possibility that the head and body movements caused by the VR content generated a lot of artifacts, which contaminated the EEG signals. In the present study, the participants sat in a chair and observed the canon and the cannonballs in front of them; thus, they did not necessarily require larger body movements to do the task. In fact, the standard error of the ERP waveforms over the participants was not large (Figure 5). However, there was no guarantee that artifacts caused by head and body movements could be completely removed from the EEG signal in the present study. To better attenuate the artifacts evoked by the head and body movements, noise removal techniques can use estimations of those artifacts to effectively denoise the EEG signals (Gwin et al., 2010). Finally, the game experience of the participants in the experiment was uncontrolled. The gaming score may vary depending on the presence/absence of the game experience of participants. Furthermore, the response to negative/positive feedbacks may change. A comparison of the ERP activities of experienced participants and naive participants who rarely play games is a topic for future experiments.

Previous studies have reported a correlation between the averaged accuracy of the entire experimental results and the modulation of the feedback potential (Maurer et al., 2015; Joch et al., 2017). However, to the best of our knowledge, no studies have reported on the relationship between the temporal accuracy for the task and the FN modulation at that time. Here, the temporal accuracy for the task was defined as the mood. We used a single-based LME analysis to reveal the relationship between the mood and FN modulations. The results suggest that FN is sensitive to the temporal mood in the game. In other words, FN might reflect flow or ambiance in the game. Because FN is modulated according to the temporal fluctuation of the mood, it gives new evidence of error-related processing in the human brain. In addition, it is worth noting the use of a single-trial-based LME analysis for the negative potentials in error events instead of the differences in the waveforms between the hit and error events. Several studies have previously treated the negative potential in the differences of the waveforms between good and bad events (dFN in this manuscript) as FN modulation. Consequently, even if the negative potential of dFN was modulated, it was difficult to determine whether the modulation was caused by positive feedback or by negative feedback. In this study, we observed that the negative potential of an error event is modulated not only by the outcome probability but also by the temporal fluctuation of the mood. This result reinforces the importance of treating the negative potential evoked by the negative feedback as FN rather than treating FN as a difference between the negative potential evoked by the error trial and the positive potential evoked by the hit trial.

## Conclusion

To summarize, this study used a VR shooting game as an experimental task and recently defined temporal mood in addition to the outcome magnitude and outcome probability. FN was observed during an error event in a shooting game and RewP was observed during a hit event. A linear mixed-effects model was used to investigate the relationship between the game factors and the modulation of the ERPs. As a result, the amplitudes of FN and RewP were changed according to the outcome probability, which is the task difficulty. Furthermore, FN was changed according to the temporal fluctuation of the mood, which is the participant’s temporal mental state in the game. We found that FN, a negative potential in negative feedback, was sensitive to temporal mood and was one of the experimental features that amplify the potential.

## Data Availability Statement

The datasets generated for this study are available on request to the corresponding author.

## Ethics Statement

The studies involving human participants were reviewed and approved by Ethical Committee for Human and Animal Research of the National Institute of Information and Communications Technology. The patients/participants provided their written informed consent to participate in this study.

## Author Contributions

YN and YY designed the experiments. YY conducted the experiments, wrote the main manuscript text, and prepared all the figures. Both authors interpreted the data and reviewed the manuscript.

## Conflict of Interest

The authors declare that the research was conducted in the absence of any commercial or financial relationships that could be construed as a potential conflict of interest.
