# Supplementary material for: Temporal Fluctuation of Mood in Gaming Task Modulates Feedback Negativity: EEG Study With Virtual Reality
Source: Front Hum Neurosci. 2021 Jun 3;15:536288. doi: 10.3389/fnhum.2021.536288 (PMC8209254; doi:10.3389/fnhum.2021.536288)
Supplement: Supplementary file 1 [file Table_1.DOCX]

Supplementary Material

**Supplementary VIDEO S1**. Video of VR shooting game. The frame rate was 30 frames per second.

**
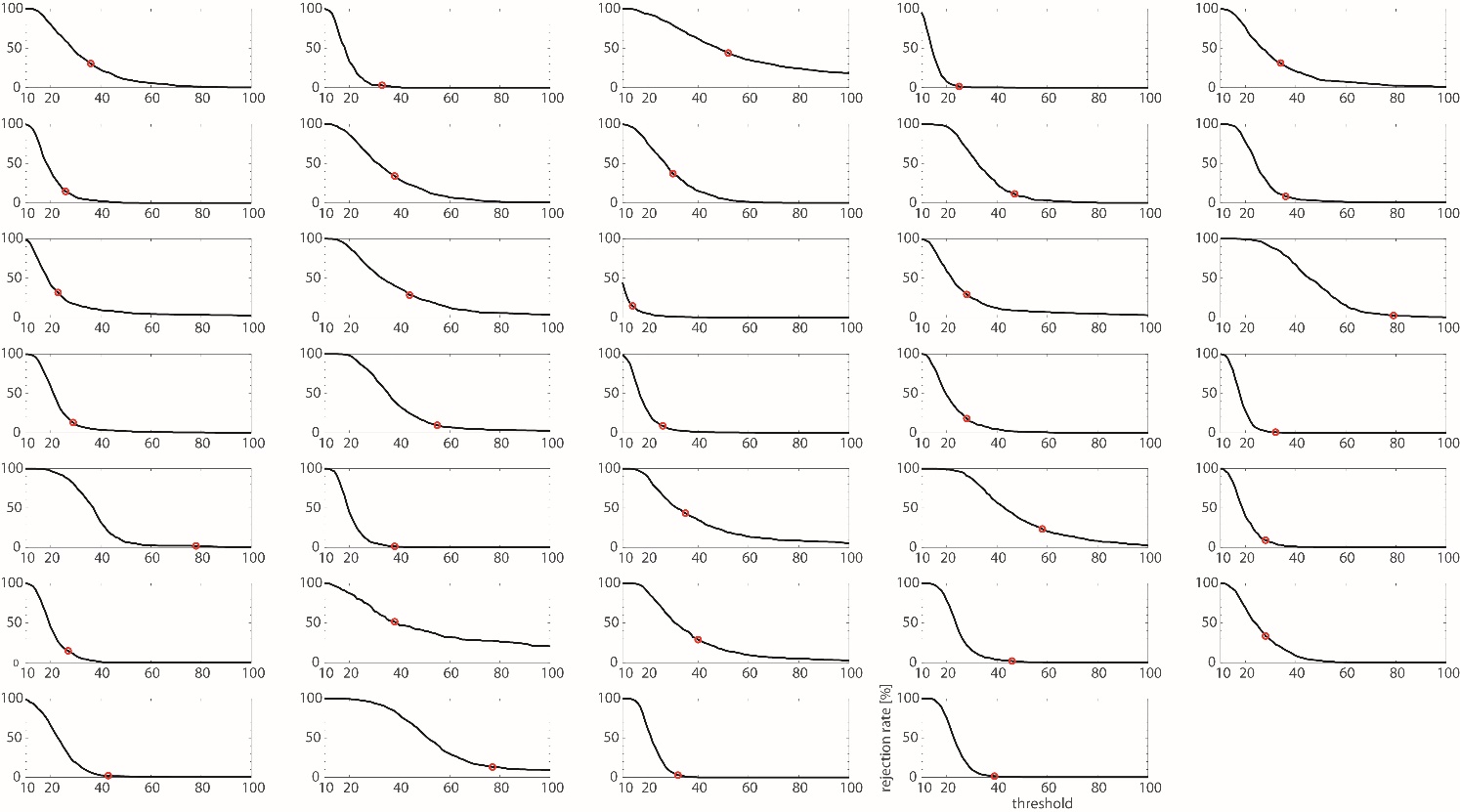
**

**Supplementary Figure 1.** Optimal threshold of each participant and rejection rate.


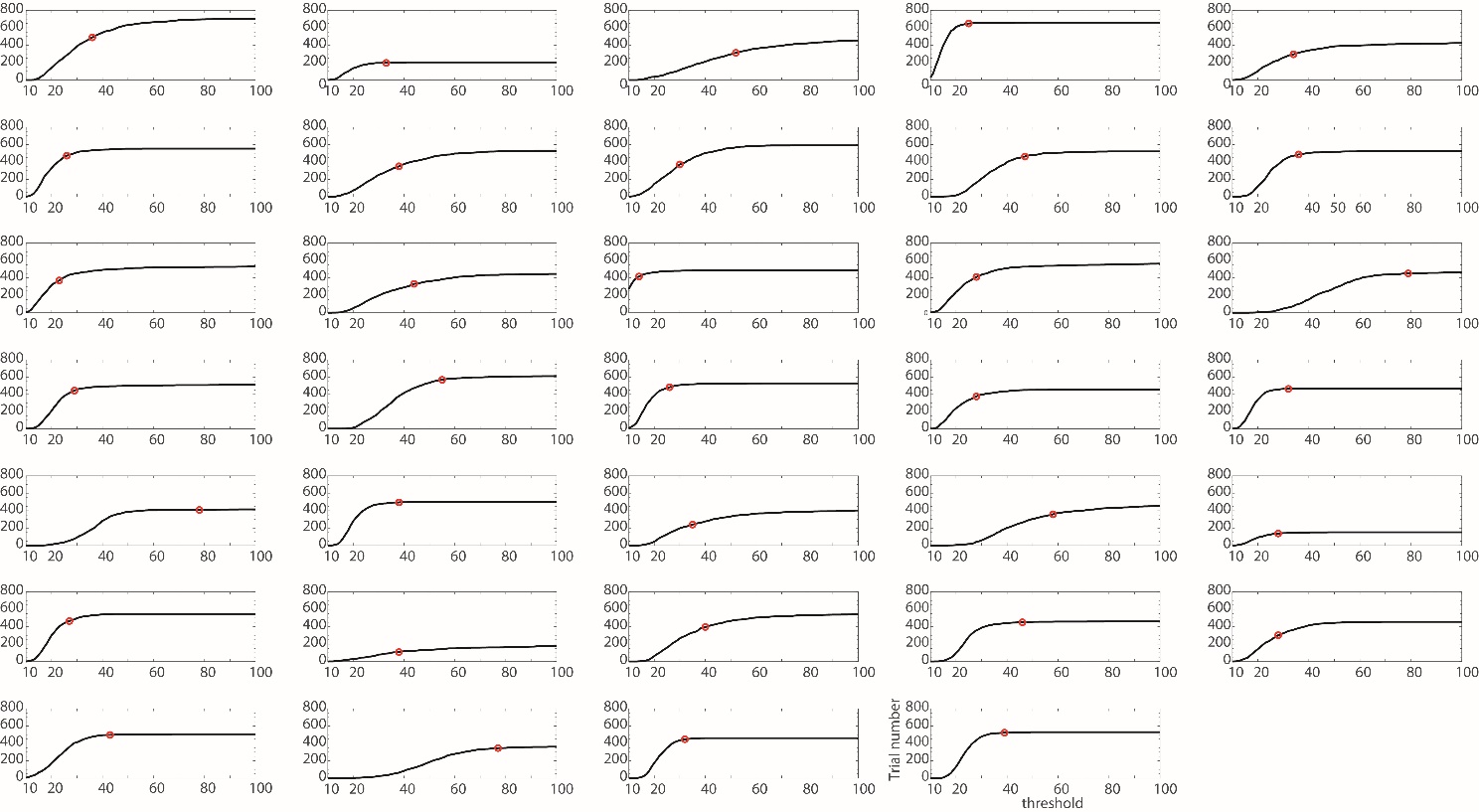


**Supplementary Figure 2**. Optimal threshold of each participant and remaining trials.
